# Fixed Prosthetic Restorations and Periodontal Health: A Narrative Review

**DOI:** 10.3390/jfb13010015

**Published:** 2022-02-01

**Authors:** Viritpon Srimaneepong, Artak Heboyan, Muhammad Sohail Zafar, Zohaib Khurshid, Anand Marya, Gustavo V. O. Fernandes, Dinesh Rokaya

**Affiliations:** 1 Department of Prosthodontics, Faculty of Dentistry, Chulalongkorn University, Bangkok 10330, Thailand; viritpon.s@chula.ac.th; 2 Department of Prosthodontics, Faculty of Stomatology, Yerevan State Medical University after Mkhitar Heratsi, Str. Koryun 2, Yerevan 0025, Armenia; 3 Department of Restorative Dentistry, College of Dentistry, Taibah University, Al Madinah, Al Munawwarah 41311, Saudi Arabia; drsohail_78@hotmail.com; 4 Department of Dental Materials, Islamic International Dental College, Riphah International University, Islamabad 44000, Pakistan; 5 Department of Prosthodontics and Implantology, College of Dentistry, King Faisal University, Al-Hofuf, Al Ahsa 31982, Saudi Arabia; zsultan@kfu.edu.sa; 6 Department of Orthodontics, University of Puthisastra, Phnom Penh 12211, Cambodia; amarya@puthisastra.edu.kh; 7 Center for Transdisciplinary Research, Saveetha Dental College, Saveetha Institute of Medical and Technical Science, Saveetha University, Chennai 600077, India; 8 Periodontics and Oral Medicine Department, University of Michigan School of Dentistry, Ann Arbor, MI 48109, USA; gustfern@umich.edu; 9 Department of Clinical Dentistry, Walailak University International College of Dentistry, Walailak University, Bangkok 10400, Thailand

**Keywords:** zirconia, ceramics, cobalt-chromium, CAD/CAM, crown, fixed partial denture, margin fit, periodontium, gingival health, gingival inflammation, gingival crevicular fluid

## Abstract

Periodontal health plays an important role in the longevity of prosthodontic restorations. The issues of comparative assessment of prosthetic constructions are complicated and not fully understood. The aim of this article is to review and present the current knowledge regarding the various technical, clinical, and molecular aspects of different prosthetic biomaterials and highlight the interactions between periodontal health and prosthetic restorations. Articles on periodontal health and fixed dental prostheses were searched using the keywords “zirconium”, “CAD/CAM”, “dental ceramics”, “metal–ceramics”, “margin fit”, “crown”, “fixed dental prostheses”, “periodontium”, and “margin gap” in PubMed/Medline, Scopus, Google Scholar, and Science Direct. Further search criteria included being published in English, and between January 1981 and September 2021. Then, relevant articles were selected, included, and critically analyzed in this review. The margin of discrepancy results in the enhanced accumulation of dental biofilm, microleakage, hypersensitivity, margin discoloration, increased gingival crevicular fluid flow (GCF), recurrent caries, pulp infection and, lastly, periodontal lesion and bone loss, which can lead to the failure of prosthetic treatment. Before starting prosthetic treatment, the condition of the periodontal tissues should be assessed for their oral hygiene status, and gingival and periodontal conditions. Zirconium-based restorations made from computer-aided design and computer-aided manufacturing (CAD/CAM) technology provide better results, in terms of marginal fit, inflammation reduction, maintenance, and the restoration of periodontal health and oral hygiene, compared to constructions made by conventional methods, and from other alloys. Compared to subgingival margins, supragingival margins offer better oral hygiene, which can be maintained and does not lead to secondary caries or periodontal disease.

## 1. Introduction

Proper treatment planning and prosthetic treatment are essential for the long-term outcome of prosthetic dental treatment. There is a strong association between prosthetic dentistry and periodontics as periodontal health has an important role in the longevity of fixed dental restorations [1,2,3]. On the other hand, defective prostheses may contribute to the progression of periodontal diseases [4]. The final finish of the prosthetic restoration also affects the development of biofilm, as increased surface roughness creates a favorable environment for microbial growth. Hence, a good prosthesis surface finish from proper manufacturing technique is important [5]. To achieve a successful treatment outcome, prosthodontists and periodontists should collaborate, to enhance the longevity of the restoration and improve periodontal health, as well as improve the quality of life for dental patients [3,6]. 

**Figure 1:**
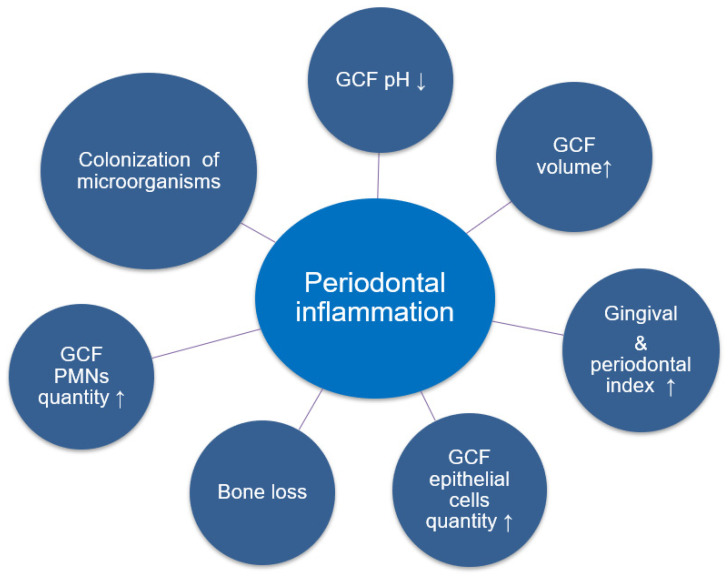
The clinical characteristics of periodontal inflammation. GCF = gingival crevicular fluid; PMNs = polymorphonuclear leukocytes.

Morpho-functional impairments of the maxillofacial complex conditioned by periodontal pathology are five times more common compared with those arising from dental caries [7]. Periodontitis is an inflammatory disease involving the periodontal tissues (cementum, periodontal ligament, alveolar bone, and gingiva) supporting the teeth [8]. It results in teeth loss requiring prosthetic treatment. A delay in prosthetic treatment causes biomechanical impairment of the stomatognathic system, worsening of the periodontal condition, and adverse consequences on the patients’ general health and behavior [9,10,11,12]. Prosthetic treatment receives special attention in the case of patients with periodontal pathology [13,14]. However, sometimes, inappropriate prosthetic treatment planning or the prosthesis itself can cause periodontitis or gingival recession and associated clinical characteristics (Figure 1).

The aim of this article is to review and present the current knowledge regarding the various technical, clinical, and molecular aspects of different prosthetic biomaterials and highlight the interactions between periodontal health and prosthetic restorations.

## 2. Materials and Method

Articles on periodontal health and fixed dental prostheses were searched using the keywords “zirconium”, “CAD/CAM”, “dental ceramics”, “metal-ceramics”, “margin fit”, “crown”, “fixed dental prostheses”, “periodontium”, and “margin gap” in PubMed/Medline, Scopus, Google Scholar, and Science Direct. Further search criteria included being published in English literature, and between January 1981 and September 2021. Then, relevant articles were selected, included, and critically analyzed in this review.

## 3. Marginal Fit and Internal Adaptation of Fixed Dental Prostheses

Marginal as well as an internal discrepancy with an external gap in a prosthetic crown/fixed partial denture are critical factors as they deal with the structural rigidity, marginal integrity, and maintenance of pulpal and periodontal health [15]. Most importantly, the position of a gap is of great importance; namely, if it is supragingivally, paragingivally or subgingivally. In gaps occurring supragingivally, better oral hygiene can be maintained and does not lead to secondary caries or periodontal disease. Subgingival gaps do not permit maintenance of good oral hygiene and should be evaluated properly. A commonly accepted approach to the optimal value of margin gap still has not been recommended. Some researchers consider the value <120 μm [16,17,18] to be optimal, while others consider that it should be <100μm [19,20]. Moreover, it is still believed that the adequate value should range between 20 and 75 μm [15].

**Figure 2:**
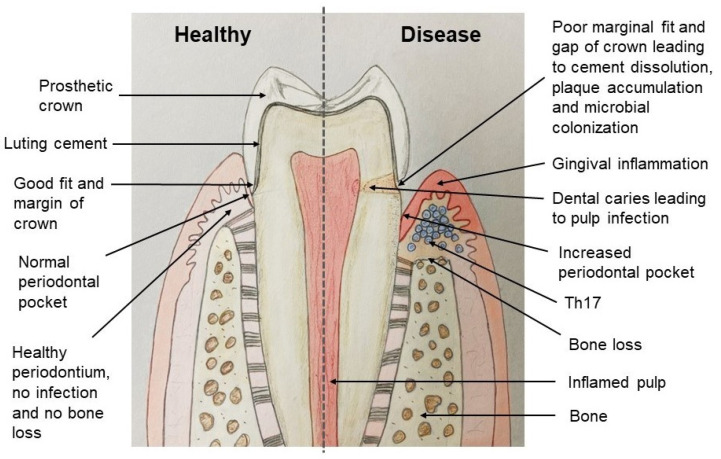
Good marginal fit of the crown and a poor marginal fit leading to consequences.

The accuracy of marginal and internal adaptation is essential for the final result and survival of a fixed prosthetic treatment. A marginal discrepancy results in thick cement, which is affected by the oral environment more, resulting in cement dissolution and deposition of dental biofilm, microleakage, margin discoloration, increased gingival crevicular fluid (GCF) flow, recurrent caries, pulp infection and, lastly, periodontal lesion and bone loss, which lead to the failure of prosthetic treatments [15]. Figure 2 shows an adequate margin fit of the crown and an inadequate margin fit leading to undesirable consequences. Hence, to protect the periodontium, especially the gingival margin and tissue-biomaterials interface, the fixed dental prosthesis should be appropriate, healthy, and durable [21].

Although no substantial difference was seen in the internal precision in the computer-aided design and computer-aided manufacturing (CAD/CAM) restorations [22], it has been shown that the internal discrepancy of digitally manufactured CAD/CAM restorations present a higher margin fit of inlays [23]. Riccitiello et al. [24] showed that both zirconia and lithium disilicate CAD/CAM prosthetic restorations provide a better marginal fit than heat-pressed lithium disilicate constructions. Another study showed no significant difference in the margin fit, both horizontally and vertically between the lost wax and CAD/CAM techniques for full coverage lithium disilicate crowns [25]. Though there are data showing a margin discrepancy in crowns made by CAD/CAM technology [26], compared with metal fused to porcelain restorations, all-ceramic constructions made by the CAD/CAM method had a higher precision [27]. Moreover, the direct digitalization of prepared teeth showed a better outcome for margin accuracy, compared to indirect digitalization, when scanning is accomplished from a conventionally molded plaster model [28]. In addition, the CAD/CAM fabricated prostheses show an improved adaptation compared to the ones fabricated using the conventional method [29,30]. Furthermore, Sorrentino et al. [31] demonstrated that zirconia copings did not show any sign of tetragonal to monoclinic transformation at the margins, irrespective of the preparation geometry.

**Figure 3:**
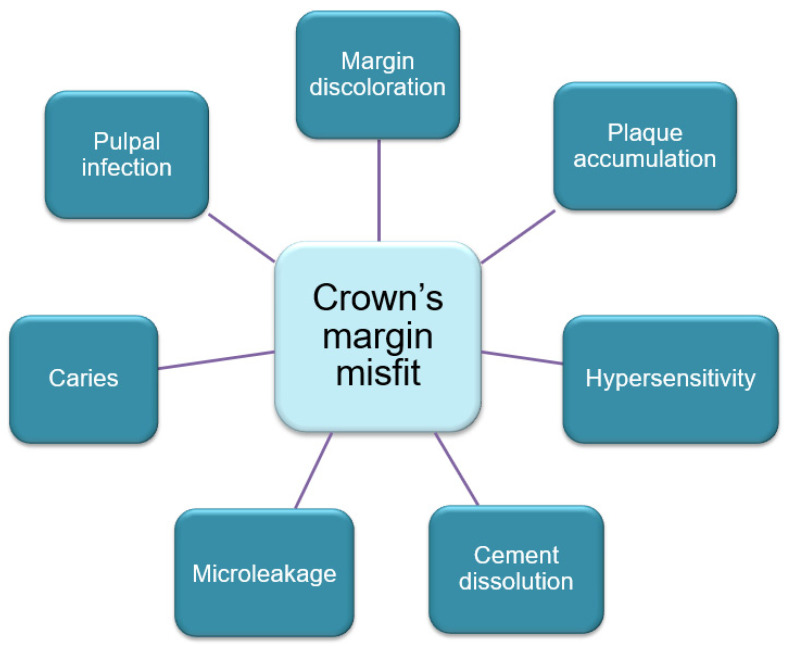
Consequences of the crown’s margin misfit.

The deterioration of periodontal tissues due to margin misfit forms a retentive region and enables the accumulation of dental plaque that occurs following prosthetic treatment with conventionally fabricated metal–ceramic restorations. The consequences of the crown’s margin misfit are shown in Figure 3. An undesirable consequence of metal–ceramic constructions on the periodontium, resulting in periodontitis, is related to mechanical trauma to the gum during tooth preparation and gingival retraction as well as with uneven contours and the topography of the crown margin [32].

The esthetics and longevity of the prosthesis are conditioned by the harmony and biofunctionality between the prosthetic construction and the periodontium [3]. The biofunctionality of a prosthesis refers to a function that is dependent on biological content. The life span of fixed prosthetic constructions depends on the periodontal status of the supporting teeth, since the mucosa in this region is subject to continuous mechanical trauma and bacterial contamination [33,34]. Thus, numerous factors, such as the condition of abutment teeth, pontic design, prosthesis construction, occlusion, and biomaterial have tangible influence and should be considered when planning the prosthodontic treatment [35]. The preservation of periodontal health around the crown’s margins is a serious challenge for a dentist, and detecting the restoration margin relative to the neighboring bone is a significant factor when providing for the long-lasting health of the gingival tissues [36,37]. The emerging couture of the prosthetic construction can affect the gingiva reaction to the fixed prosthesis. Moreover, the life span of fixed dental restorations is governed by the margin adaptation of the prostheses [15].

An incorrectly fabricated dental prostheses construction may harm both the health of the oral tissues and worsen any existing periodontal pathology. Rough and irregular surfaces on the restorative biomaterials may create a favorable environment for microbial invasion and biofilm formation [38,39,40]. Any rough and irregular margins on the fixed prosthetic constructions can also result in microorganisms’ attachment. The aforementioned factors can worsen oral hygiene conditions and cause gingivitis and further periodontitis. Zirconia surface relief alterations and roughness following the application of laser irradiation were studied by Popa et al. [41]. The group Nd:YAG consists of zirconia restorations treated with a neodymium laser and the group Er:YAG included restorations treated with an erbium laser. The authors noted that there were substantial differences between the Nd:YAG and Er:YAG following surface irradiations. The Nd:YAG laser shaped more changes in the zirconia surface than the Er:YAG. Thus, poor oral hygiene was detected in 35% of the patients with conventionally fabricated metal–ceramic dental prostheses, in 30.3% of the subjects with CAD/CAM fabricated metal–ceramic prostheses, and 28.6% of the patients with zirconia-based ceramic prostheses, one year after the insertion of the constructions [42]. In addition, fixed prosthetic restorations, such as crowns and bridges may interfere with the host’s defensive mechanisms creating regions of microbial colonization and resulting in bacterial biofilm formation, which subsequently damages the periodontal tissues [43]. 

## 4. Biologic Width and Gingival Biotype Considerations in Fixed Prosthetic Restorations and Periodontal Health

Biologic width is a natural seal that is present around the teeth, protecting the alveolar bone from infection and diseases [44]. The biological width is defined as the dimension of the soft tissue that is attached to the portion of the tooth coronal at the crest of the alveolar bone [45].

Gargiulo et al. [46] described the dimension of biologic width as consisting of a sulcus depth of 0.69 mm; junctional epithelium of 0.97 mm (0.71–1.35 mm); and supra-alveolar connective tissue attachment of 1.07 mm (1.06–1.08 mm). So, the biologic width is commonly stated to be 2.04 mm, representing the epithelial and connective tissue measurements.

The biologic width is an essential space that must be maintained to ensure periodontal health in any dental prosthetic restorations [47]. Hence, it is important to preserve the periodontal health and remove any irritation that might damage the periodontium such as may occur during prosthetic restorations [45,48]. Considering this, an iatrogenic fixed dental prosthesis that is constructed in violation of the biologic width predisposes the development of subgingival caries in the involved teeth and results in an uncontrolled inflammatory process and periodontal tissue destruction [47]. 

Nevins and Skurow [49] mentioned that, in cases where the subgingival margins are indicated, the dentist should not disrupt the junctional epithelium or connective tissue during tooth preparation and taking an impression. They also suggested limiting the subgingival margin extension to 0.5–1.0 mm, as it is impossible for the dentist to detect where the sulcular epithelium ends and the junctional epithelium begins. 

In addition, the crown placement margins affect gingival health, especially in subgingival areas. Reitemeier et al. [50] studied the effect of posterior crown margin placement on gingival health. The variables were: alloy used, location of crown margins, oral hygiene index score, plaque index, and sulcus bleeding index scores. They found that the lingual surfaces showed the highest probability of plaque compared to the facial surfaces. The risk of gingival bleeding at the posterior crown margins was approximately twice that seen at the supragingival margins. Poor oral hygiene before treatment and the presence of plaque were also associated with sulcular bleeding. The type of alloy did not influence sulcular bleeding. The probability of plaque at 1 year increased with an increasing oral hygiene index score before treatment. In addition, Ercoli and Caton [51] mentioned that the restoration margins placement within the junctional epithelium and supracrestal connective tissue attachment can be associated with gingival inflammation and, potentially, recession [51]. The presence of the fixed prostheses finish lines within the gingival sulcus does not cause gingivitis if the patients are compliant with self-performed plaque control and periodic maintenance.

The gingival morphology is partially related to the tooth form and shape. Tooth shapes can be square, triangular, or ovoid [52]. Patients with square-shaped teeth have more favorable esthetic outcomes due to long proximal contacts and less papillary tissue, whereas patients with triangular-shaped teeth have a proximal tooth contact located more incisally and, thus, more tissue height to fill in and a high risk of gingival recession and black triangle occurrence [53].

Likewise, overlooking the biotype of the gingiva when planning a fixed dental construction could exacerbate the existing pathology [54,55]. For instance, the gingival crevice depth and the gingival tissue thickness (biotype), as well as the alveolar crest location, vary among patients and need consideration during the treatment [56,57]. It was found that the gingival biotype plays an important role in the treatment result [6,21,58,59]. Having knowledge of the characteristic features of gingival biotypes can help to minimize tissue resorption and provide improved results in both tooth preparation and gum recession. Biological width violation, as well as inappropriate tooth preparation, may lead to an alteration in soft tissue thickness, transforming it into thin gingiva over time. It was confirmed that the thick gingiva may undergo alteration to a thin gingival biotype over time in response to prosthetic treatment [41]. These thin, soft tissues have a higher predisposition toward recession, which necessitates the supragingival placement of restoration margins wherever possible.

León-Martínez et al. [60] studied the periodontal behavior of teeth prepared with horizontal finishing crowns supporting fixed metal-ceramic, zirconia full-coverage crowns, and fixed partial dentures. In the control teeth, they found higher plaque control and bleeding upon probing, as well as probing pocket depth; whilst probing attachment levels were higher around the teeth prepared with horizontal finishing lines supporting complete coverage crowns and fixed partial dentures. Gingival migration was seen in periodontally compromised teeth prepared with horizontal finishing lines. They concluded that the teeth prepared with horizontal finishing lines supporting crowns and fixed partial dentures present more periodontal disorders than untreated control teeth.

Another useful technique is the tooth preparation margin, a technique designed to create an anatomic crown with a prosthetic emergence profile, which simulates the shape of the natural tooth; it is also known as the biologically oriented preparation technique (BOPT) [61,62]. This is a prosthetic technique for periodontally healthy teeth, which uses a feather edge preparation in a flapless approach in both esthetic and posterior areas with fixed prosthetic restorations, achieving high quality clinical and esthetic results in terms of soft tissue stability at the prosthetic–tissue interface [63]. In addition, the BOPT technique is simpler and faster during preparation impression taking, temporary crowns’ relining, and creating the crowns’ profiles, up to the final prosthetic restoration when compared to chamfer, shoulder, etc.

Agustín-Panadero et al. [61] studied the clinical behavior of crowns and fixed partial dentures on teeth using a vertical preparation without finish line BOPT. They found that two years after treatment, the vertical preparation without finish line BOPT produced gingival thickening, margin stability, and optimal esthetics. Neither crowns nor fixed partial dentures showed any mechanical complications.

## 5. Gingival, Periodontal, Hygienic Indexes, and Clinical Manifestations

The consequences of fixed prosthetic constructions fabricated by different biomaterials and technologies on healthy and pathological periodontium were investigated by Avetisyan et al. [21]. The missing teeth of patients with partial edentulousness were recovered with either conventional cobalt-chromium (Co-Cr) based, CAD/CAM Co-Cr based, or CAD/CAM zirconium dioxide-based ceramic prosthesis. The oral health condition together with the periodontium was evaluated before and after the placement of the prosthetic restorations using different periodontal and hygienic indexes, such as the modified approximal plaque index (MAPI) and the community periodontal index (CPI). Additionally, the gingival biotype was determined using the probe transparency technique. After 12 months of prosthetic treatment, the mean value of MAPI stayed practically unchanged in the patients with diagnosed periodontitis with both conventionally manufactured Co-Cr-based, and CAD/CAM Co-Cr-based ceramic restorations. Furthermore, zirconia-based ceramic constructions demonstrated better periodontal outcomes, decreased inflammation, and improved oral hygiene conditions. In addition, the individual periodontal biotype should be considered before prosthetic rehabilitation to avoid periodontal tissue trauma and to prevent the colonization of microorganisms [20].

A study by Abduo and Lyons [64] stated that there is no direct association between the status of the periodontium and the longevity of fixed dental restorations. However, harmony between the periodontium and the prosthetic construction is imperative. Otherwise, the esthetics and the survival of the restoration will be compromised. The gingival tissue reaction to the prosthetic construction is conditioned by the position of the finish line, the couture, and the emergence profile of the restoration. The clinical and esthetic results as well as the gingival tissue reaction are also associated with the restoration cleanability and pontic design. Even if the pontic design is adequate, it cannot preclude the development of mucosal inflammation near to the pontic if the oral hygiene conditions are not preserved via dental biofilm elimination. The patients’ capability to accomplish optimal oral hygiene is indispensable for the survival of the prosthetic construction, and systematic checkups provide a chance for the early recognition and management of complications.

The fixed prosthetic constructions may cause inflammation, and when it becomes chronic, the adaptive mechanisms of immunity are stimulated, involving cellular and non-cellular immunity. These immune mechanisms have a critical role for the further limitation of the inflammatory reaction, and in the recovery process with the regeneration and the restoration of injured tissues. Thus, self- and acquired immune mechanisms should be synchronized to return the damaged tissue to homeostasis [65]. Ercoli and Caton [66] noted that the accumulation of plaque and loss of periodontal attachment is related to the type of prosthetic restorations. The margin of restoration located near the junctional epithelium can cause periodontal inflammation and gingival recession.

The early development of the lesion takes place as a reaction of local leukocytes and endotheliocytes to the dental plaque around the prosthetic restoration margins. The metabolic byproducts of these microorganisms activate junctional epitheliocytes, promoting cytokines and stimulating neuropeptides release, which leads to the dilatation of blood vessels. With the development of the pathological process, increased numbers of various cells such as neutrophils, macrophages, plasma cells, lymphocytes, and mast cells migrate towards the pathological foci. When the pathological foci are formulated, a transformation from the self- to the acquired immune response occurs. Plasma cells and macrophages, as well as B and T lymphocytes prevail; IgG3 and IgG1 subtypes of B lymphocytes also exist. Blood flow disturbance, as well as collagenolytic activity amplification, is also observed. There is also an amplified collagen production by fibroblasts. This clinical phase is accompanied by gingival bleeding, gingival color, and contour alterations, and is assessed as moderate to severe gingivitis. Clinically, the advancement of lesions results in the development of periodontitis. In this stage, irreversible periodontal attachment and alveolar bone loss are detected, clinically and histologically. With the advancement of inflammation, periodontal pocket development occurs [65,67].

The most common complaints among subjects following prosthetic treatment using conventionally manufactured metal–ceramic restorations were the occurrence of discoloration of the gingival papilla, a dark shade around the restoration margin edges, and development of gingivitis. Nevertheless, these clinical symptoms were absent when metal–ceramic restorations were made using the CAD/CAM technology, which is possibly due to an improved margin adaptation of the metallic base [19]. All of these signs were absent in the subjects who received zirconia-based ceramic constructions as the zirconium does not affect soft periodontal tissues and, instead, stimulates the protective mechanisms of the periodontium. Additionally, clinical recovery was noted at the margin of zirconia-based ceramic constructions. All mentioned properties are conditioned by less microbial adhesion to zirconium when compared with base metal [68,69].

Subjects with fixed prosthetic restorations made by the CAD/CAM technology had improved periodontal response in comparison with conventionally manufactured fixed dental constructions [70,71,72]. These fixed restorations perform in an extremely complex oral environment with uncontrolled elements such as masticatory load, temperature, and pH changes. Thus, the performance of the prosthetic construction may be affected by the biomaterials, fabrication technologies, operator skills, or host-related factors. 

Before the prosthodontic treatment, no significant changes were found in the periodontal index scores among subjects with various fixed dental restorations. Long-term outcomes concerning the effects of the dental constructions based on the applied biomaterials and technics on periodontium were observed in patients 1 year after the prosthetic treatment. A significant variation was established in the subsequent parameters: healthy sextants quantity, which was higher among subjects with periodontitis who received zirconia-based ceramic restorations compared with those with Co-Cr-based ceramic groups. Likewise, the number of sextants with 4–5 mm periodontal pockets was lower in the subjects with periodontitis who received zirconia-based ceramic restorations compared with a conventionally fabricated Co-Cr-based ceramic group. Statistical differences regarding the existence of clinical symptoms such as the hygienic index, bleeding, 6 mm or more periodontal pockets, as well as excluded segments, were not detected amongst all of the subjects, which were also approved by the medical examination [21].

The gingival health and oral hygiene condition in subjects following the insertion of fixed prosthetic constructions were investigated by Basynet et al. [73]. Various factors, such as the prosthetic construction type (fixed partial denture, single crown) and biomaterial (metal, metal–ceramic) are statistically related to the gingival condition and oral hygiene. The gingiva and plaque index [74] was used to examine the teeth and gingiva. The analyses were performed after 2 weeks and 6 months following the insertion of the prosthetic construction. No difference was established in the plaque index among the subjects who received crowns, whereas those with a fixed partial prosthesis presented with significance. The results for the type of biomaterial were not significant. Statistically, similar outcomes for the gingival index were displayed. The authors established that the prosthetic crown had no substantial influence on the gingival and plaque indexes of the subject after 2 weeks and 6 months, whereas the bridges exhibited a considerable effect. Regardless of the dental material (metal or metal–ceramic) no differences were found on the gingival and plaque indexes for crowns [68]. The necessity for plaque control and oral health education programs is needed for the fixed dental prostheses to decrease the occurrence of periodontal pathology. Moreover, it was shown that fixed dental restorations of various types affect gingival and periodontal health [21]. The latter statement is consistent with other studies [66,75,76].

Patients with inflamed periodontal tissues demonstrate periodontal index changes and clinical symptoms of inflammation (discomfort, bleeding of various severity, gum tenderness, and halitosis) [77]. A fixed prosthetic restoration may worsen the periodontal status of the mucosa under the pontic if the hygienic condition is not preserved via plaque elimination. Therefore, the patients’ compliance regarding the preservation of good hygienic conditions is essential for the survival of the dental construction [64,78].

Al-Sinaidi et al. [76] evaluated the periodontal health in subjects who received fixed dental restorations, and the outcomes of subgingival and supragingival located crown margins were also evaluated. The authors noticed higher gingival and plaque indexes as well as a deeper periodontal pocket in the abutment teeth compared with non-abutment teeth. Additionally, supporting teeth with higher gingival and plaque index scores as well as probing pocket depth presented in patients who had their functioning fixed dental restorations for approximately 5 years, as well as those who were older than 46-years old. The teeth with subgingival positioned crown edges had considerably lower mean values for their clinical parameters than the teeth with supragingival crown edges.

When dental prostheses demonstrate greater biofilm buildup and increased inflammatory levels, this suggests that additional measures are required to control these factors [79]. These measures can be mechanical and chemical control aids. Mechanical control consists of toothbrushes (manual or electric) and toothpaste as well as specific devices for interdental cleaning. In addition, the chemical agents (antiseptics) exhibit antimicrobial benefits when used for prosthesis disinfection, though only a few agents can be used safely without causing damage. The most common chemical agents consist of three types of mouthwash with antiplaque and antigingivitis effects, which are chlorhexidine (CHX); essential oils (EOs), ranked second; and cetylpyridinium chloride (CPC) [80,81]. The CHX solution (0.2%) is the most indicated [81]. EOs are the second choice as they are not as effective as the CPC solution (0.05% to 0.75%), which, however, has a greater potential for adverse reactions [80]. The adverse effects of mouthwash includes a transient loss of taste sensation, bitter taste, soreness and burning sensation of the mucosa, dryness of the mucosa, and epithelial desquamation [81]. Fluoride solutions can be used for high caries risk patients.

## 6. Cytomorphometric Analysis Following Fixed Dental Prosthesis

Cytomorphometric analysis is regularly performed in numerous diagnostic procedures [82]. This method, which uses exfoliative cytology, is quite fast, straightforward, and trustworthy, and permits the repetitive collection of biological substances without influencing the integrity of the local tissues. Subsequently, the present method can be accomplished repeatedly in screening programs as well as in the course of dental examinations [83]. Nevertheless, the cytological method for the detection of periodontal pathology was not defined appropriately until now, and it is infrequently used in dental and periodontal practice [84,85]. The GCF cytomorphometric analysis aids in the identification of the cellular structure and periodontal pocket composition in subjects with fixed prosthetic constructions. Alterations in periodontal tissues occur during prosthetic treatment with fixed prosthetic constructions, which take place during the initiation of the adaptive and regenerative processes [86].

Recently, inflammation dynamics in the periodontium was studied by Heboyan et al. [77] using the cytomorphometric method before and after the insertion of fixed prosthetic restorations (Co-Cr-based ceramic restorations made by the conventional technique as well as the Co-Cr and zirconium dioxide-based ceramic constructions made using the CAD/CAM technology) among healthy patients and patients with periodontal pathology. The authors concluded that, irrespective of the restoration type applied, no substantial alteration in the parameters was recognized among healthy subjects, before and after 1 year of prosthodontic treatment. Among all of the observation groups, the oral epitheliocyte quantity considerably increased while the polymorphonuclear leukocyte (PMNs) count significantly reduced following the insertion of the prosthetic constructions. Nevertheless, the CAD/CAM restorations showed an improved periodontal response. The records were similar to studies reported by other researchers [85,87].

Flat epitheliocytes and PMNs are the main constituents of the cellular scatter of gingival cytograms. The existence of these cells was observed in healthy patients and subjects with periodontal pathology. With the development of inflammation in the periodontal tissues, the quantity of PMNs, which are considered harmful to the periodontium, increased in all groups. The GCF sample analysis for the subjects with different fixed prosthetic restorations showed cytomorphological variations that were possibly associated with the fabrication technology and the biomaterial used in the dental restorations. The increased amount of PMNs in the GCF smear samples demonstrated an alteration in vascular permeability of the tissues in contact with periodontal microorganisms and the existence of an inflammatory reaction among the patients with periodontal pathology, which was also accompanied by a reduction in oral epitheliocyte count [86].

CAD/CAM fabricated constructions display improved periodontal reaction. Several CAD/CAM ceramics (e.max CAD HT, Empress CAD; e.max CAD LT; and Mark II) were investigated on human oral keratinocytes (HOK), cell viability, adenylate kinase, and excretion of human gingival fibroblasts (HGF) [71]. The authors stated that there were no substantial differences in the migration ability and cell viability of HGF and HOK on the CAD/CAM all-ceramic biomaterials. On the contrary, the conventional cast alloys for making metal–ceramic restorations are composed of metals such as Co, Ni, Cr and accompany multiple biocompatibility issues that influence the final result [72,88,89]. The existence of saliva or electrolytes may produce corrosion byproducts from the various ions from the cast alloy in tissues, which may modulate the immune system [90,91]. Ni released from Ni-containing alloys release Ni ions, which causes various reactions [92,93]. For instance, Ni-free alloys are less vulnerable than Ni-containing alloys [88,92]. Zirconium is an extremely biocompatible ceramic biomaterial and is stable in the corrosive oral environment [94,95,96]. Shang et al. [96] found that interleukin-6, TNF-α, the bleeding index, and the probing depth were all considerably high in nickel-chromium (Ni-Cr) based ceramic constructions but not in the CAD/CAM fabricated zirconia. These indicate that the all-ceramic and zirconia restorations are favorable to the health of the periodontium.

The alteration in the leukocyte-epithelial index is associated with the clinical signs of the pathology. Certainly, as the pathology develops, the PMNs are the primary protectors, and these typical modifications were detected in the GCF cytograms. The PMNs exist while fixing various types of prosthetic constructions, but their quantity was diverse. Better cytomorphometric data were noted among the subjects with periodontal pathology, depending on the biomaterial of the dental constructions [86,96]. 

**Figure 4:**
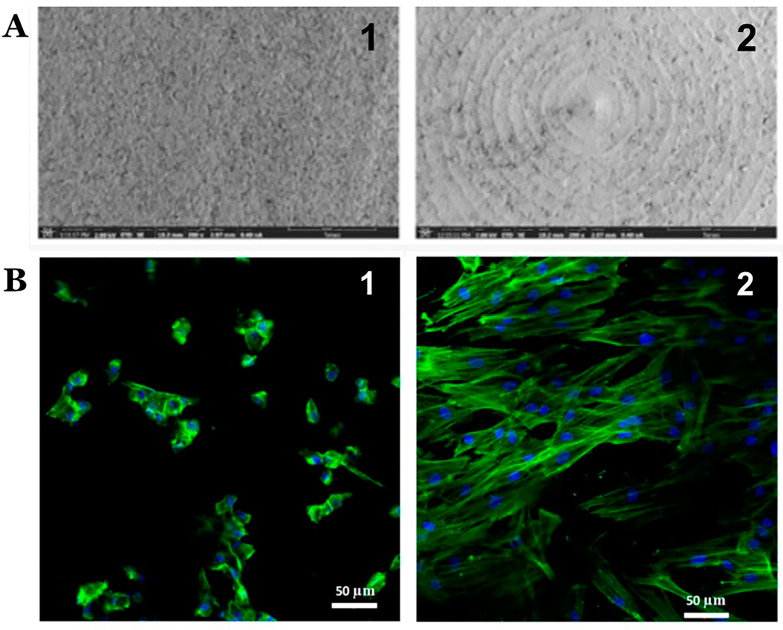
Images of surface structure using scanning electron microscopy (at 200× magnification) of ZLS (**A1**) and Y-TZP (**A2**); and confocal microscope images (20×) of fibroblasts on ZLS (**B1**) and Y-TZP (**B2**) [100].

Numerous studies have demonstrated the cytological composition of the GCF in periodontitis for diagnostic purposes [65,97,98,99]. Rizo-Gorrita et al. [100] studied the surface, cellular proliferation, and cellular morphology between Yttrium-stabilized tetragonal zirconium (Y-TZP) (VITA YZ^®^ T, VITA Zahnfabrik, Postfach, Germany) and zirconia reinforced lithium silicate ceramics (ZLS) (Celtra^®^ Duo, Degudent, Hanau-Wolfgang, Germany). They found that the ZLS surface has a porous, non-homogeneous, irregular structure with crater-like areas and random distribution, while Y-TZP demonstrated a concentric, parallel grooved pattern, resulting from the disc manufacturing process. In addition, higher proliferation and spreading were seen on the surface of Y-TZP (Figure 4). This indicates that Y-TZP is a better transgingival implant material.

It has been found that there is a lack of comparative records concerning the alteration in the GCF parameters during prosthetic rehabilitation using fixed restorations manufactured from different biomaterials [100]. Without preventive measures, inflammatory processes in the periodontal tissues might cause additional tissue damage resulting in untimely tooth loss [101,102,103]. Pathological advancement may occur as a result of oral keratinocyte reactions to the existence of periodontal lesions [104,105,106]. In a healthy periodontium, oral keratinocytes are continuously shed and exchanged by basal progenitors inside the gingival crevice. Particularly, the rate of transformation increases as the pathology advances [107,108,109]. Moreover, the PMNs are abundant in the gingival pockets among subjects with periodontal pathology. It was reported that 47% of the cells of the gingival crevice were leukocytes, with 98% being PMNs. Particularly, the total quantity of cells increases as the inflammation advances with the differential quantity of PMNs (95–97%), mononucleocytes (2–3%), and lymphocytes (1–2%) [65,110].

## 7. Salivary pH and GCF pH on Gingival and Periodontal Health

The normal pH of saliva is 6.7 (6.2–7.6), which is close to neutral, and the resting pH of the mouth does not fall below 6.3 [111]. There is an association between saliva pH and gingivitis and periodontitis. Plaque bacteria take calcium compounds and use the minerals to protect them from the high pH [111,112]. The two key factors of plaque formation are as follows: firstly, there must be oral bacteria to attack food particles and increase the pH; secondly, the pH must elevate to above 7.6 in order to grow the dental plaque crystals that cause periodontal disease. The saliva contributes to the maintenance of the pH via two mechanisms [111]: firstly, the flow of saliva removes the carbohydrates that are metabolized by bacteria and remove the acids produced by bacteria; secondly, the saliva neutralizes the acidity produced by drinks, foods, and bacterial activity, by the buffering activity of saliva.

GCF is an environment of organism with a complex composition consisting of leukocytes, desquamated epithelial cells, microorganisms, electrolytes, proteins, enzymes, and other substances [72]. The normal pH of GCF is a more alkaline 7.5–8.7 [113]. One of the most significant factors of the GCF is the pH, which is of great importance to providing an optimal environment for metabolic processes. The amount of secreted gingival fluid is known to serve as an objective criterion to assess the condition of periodontal tissues. It is an informative indicator among other diagnostic tests to determine the presence and severity of the inflammation in periodontal diseases [72,73]. 

Thus, alkaline pH is essential for plaque growth, as suggested by the mildly alkaline pH of the saliva obtained from the subjects with generalized chronic gingivitis. In addition, there is a correlation between the pH level and the microflora in periodontal pockets [114] and it is found that the periodontopathogens grow at a mildly acidic pH [115,116]. Hence, salivary pH in patients with chronic generalized gingivitis is more alkaline than that in patients with clinically healthy gingiva. In patients with chronic generalized periodontitis, the salivary pH is more acidic [111]. More studies are needed to study the physiology.

The qualitative and quantitative assessment of the GCF can be an indicator of the periodontal status, and its examination helps in the diagnosis of oral diseases [117,118,119,120]. The examination of the GCF can be a diagnostic tool to discover host-microbial communications and to reveal the phase of periodontitis [121,122,123].

Heboyan et al. [42] studied the peculiar features of the GCF parameter dynamics among subjects with fixed prosthetic constructions made using different biomaterials and manufacturing techniques. The results of studying the amount of the excreted GCF in subjects before the restoration of their masticatory function via dental prostheses of various biomaterials and manufacturing techniques, as well as after prosthetic treatment, found that the amount of excreted GCF increased 1.38-fold following restoration using the conventionally fabricated cobalt-chromium-based ceramic prostheses. Furthermore, the greatest amount of GCF is excreted in the first 6 months following the insertion of the prosthesis. On the contrary, a decrease in the excretion of GCF volume is observed between 6 months and up to 1 year. An approximate 2-fold reduction in GCF volume was observed in subjects with CAD/CAM fabricated restorations 12 months after prosthetic treatment. The reduction in this value occurs progressively and approaches a value within the normal range in the later period of up to 1 year. This tendency was noted in all prosthetic groups. The results received upon studying the dynamics of the GCF volume before and after the restoration of masticatory function found that the largest improvement in indexes is noted among patients with zirconia-based dental prostheses, as well as with Co-Cr-based ceramic prostheses fabricated by CAD/CAM technology. An insignificant GCF volume increase seen a year after the insertion of a fixed metal–ceramic restoration among certain subjects can be explained by poor oral hygiene, which influenced the statistical data of all observation groups.

Furthermore, when the margins of the artificial crown are placed subgingivally, the tissues of marginal gum are subjected to more noticeable inflammation. According to the data, oral hygiene improves during the process of prosthetic treatment [21], with GCF pH, and periodontal status changing [77,124] for the better. The study on the pH of GCF found that following the restoration of masticatory function by dental prosthesis of various frames and manufacturing techniques showed an increase in the pH index when compared to the data obtained before the prosthetic treatment. The preventive measures lead to the normalization of oral fluid pH due to the improvement of metabolic processes within the oral cavity.

## 8. Bacteriological Evaluation Following Dental Prostheses

Oral microbiota and bacterial associations of the GCF among subjects with fixed prosthetic restorations fabricated by different materials and manufacturing methods were assessed by Heboyan et al. [124]. The observation of the clinical picture showed a diversity in prosthetic treatment and presented some peculiarities depending on the nature of the dental biomaterials and technologies used for production. Among the oral pathological microflora, *Porphyromonas gingivalis* demonstrated an exceptional capability to coaggregate with *Fusobacterium* spp. and with primary invaders, such as *Streptococcus* spp. [125,126]. This clarifies its initial existence in formulating dental plaque [127], which is commonly released from the periodontal pockets among elder subjects with periodontal pathology. The existence of *Porphyromonas gingivalis* is related to an increase in cytokine release by defensive local cells [128,129]. Presenting a minor element of subgingival microflora, it considerably impacts the microbiome, devastating self-immunity pathways. Gram negative anaerobic microorganisms such as *Fusobacterium nucleatum* (*F. nucleatum*) have a central role in biofilm development, providing a connection between early and late invaders and forming the structure of the biofilm, and consequently, improving the adhesive properties of more of the microbes related to periodontal pathology [130,131]. Both *P. gingivalis* and *F. nucleatum* are also capable to join and enter into the host epitheliocytes and initiate the immune-inflammatory process of the host. *Peptostreptococci* are associated with other microorganisms and considered pathogens of mixed infections.

Generally, most microbes of the oral microbiome are symbionts that sustain a high degree of homeostasis and the healthy condition of the mouth [132]. The vast majority of oral microbial species are saprophytes and not harmful to the host. Although various microbes of biocenosis alter frequently in different regions of the human body, each person typically has more or less specific bacterial populations. The challenges in the microbiologic differentiating of *Bacteroids* in healthy and in different pathological situations of the oral tissues do not permit the recognition of a definite causative agent of the pathological process [124].

The differential microbial species and metabolites in the GCF among those with periodontal pathology and healthy subjects are potential biological indicators, indicating a possible approach to forecast, diagnose, and accomplish individualized periodontal treatment [133,134,135,136]. Periodontal pathology is related to misbalanced homeostasis in the oral tissues, microbial progression, and development of the dental biofilm on the dental prostheses. However, data on periodontal pathogens relative to periodontitis and in subjects with healthy periodontium are insufficiently presented. Moreover, the inter-relationship between host and microorganisms, as well as biochemical metabolic processes, has not been acknowledged [137,138]. 

The colonization of the gingival crevice with various microorganisms was observed at various fixed prosthetic restorations. Candida albicans noted in smear samples resulted in an inflammatory reaction in the periodontium when using Co-Cr-based ceramic dental restorations of the conventional fabrication technique, which possibly related to the weakening of the immunity in the gingival crevice. The same picture was revealed when fixed Co-Cr-based ceramic restorations were made using the CAD/CAM technique; there was a difference in the microflora number in the subjects who received the conventionally fabricated Co-Cr-based ceramic dental restorations. Zirconia-based ceramic restorations showed the best outcomes both in the qualitative and quantitative composition of microbiota in the gingival crevice. After 12 months of prosthetic rehabilitation with cobalt-chromium-based ceramic and zirconia-based ceramic fixed full coverage restorations, a quantitative reduction in various microorganisms, such as *Prevotella intermedia, Streptococcus haemolyticus*, *Porphyromonas gingivalis*, *Fusobacterium* spp., and *Corynebacterium anaerobium* was observed [124].

It is worth mentioning that *Candida albicans* were found during the bacteriologic and bacterioscopic analyses of subjects with fixed prosthetic restorations, within 1 year of follow-up, which reliably confirmed their existence. Candida albicans is also presented in normal microbiota in the form of saprophytes. Hence, the qualitative and quantitative elements of fungal pathology can be exposed only via bacterioscopy. Recent research showed the existence of pathological reactions in identifying more than six well-stained *pseudomycelia* [124].

**Figure 5:**
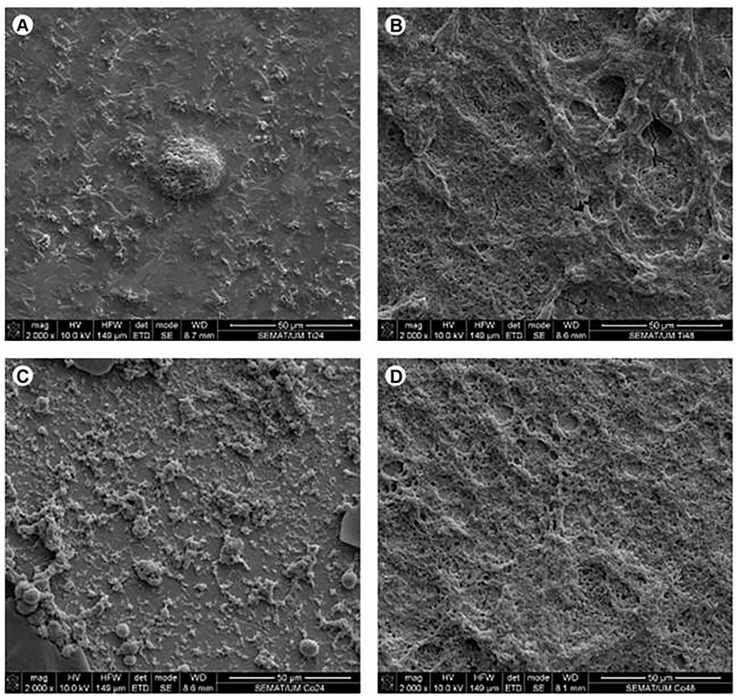
SEM figures received on commercially pure Ti surfaces (**A**,**B**) and Cr-Co-Mo alloys (**C**,**D**) covered with biofilms after 24 h (**A**,**C**) and 48 h (**B**,**D**) of growth in a BHI medium supplemented with 5% sucrose. Figures obtained using secondary electrons mode (SE) at 10 kV [139].

**Figure 6:**
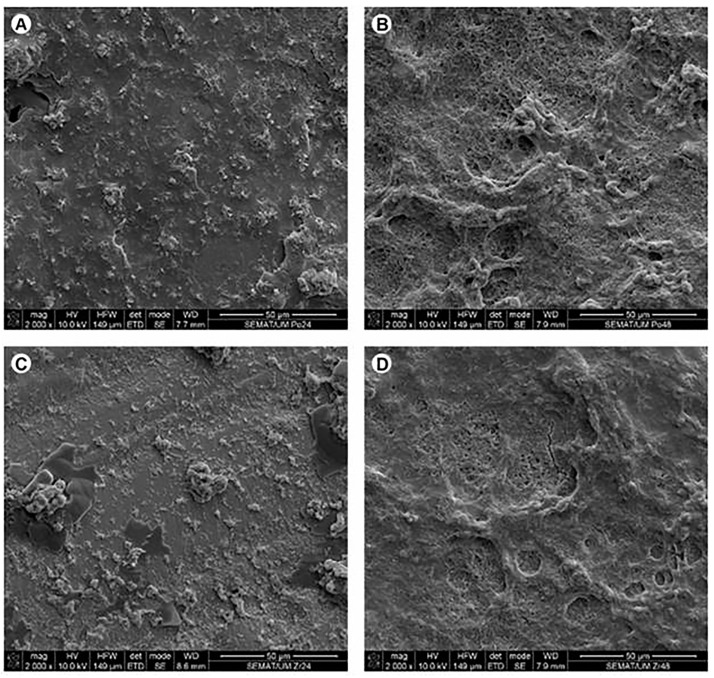
SEM figures received on feldspar-based ceramics (**A**,**C**) and Zr (**B**,**D**) covered with biofilms after 24 h (**A**,**B**) and 48 h (**C**,**D**) of growth in a BHI medium supplemented with 5% sucrose. Figures obtained using secondary electrons mode (SE) at 10 kV [139].

Mechanical preparation of the teeth is found to be harmful and to cause the worsening of periodontal status if not done properly. Nevertheless, after 1 year of prosthetic rehabilitation with fixed prosthetic constructions, periodontal recovery occurs among patients who received constructions made using the CAD/CAM technique. Additionally, a quite noticeable transformation towards clinical healing was revealed among patients with zirconia restorations because zirconium is less harmful to the periodontal tissues due to the reduced risk of dental biofilm accumulation. Additionally, zirconium is more biocompatible and tolerable to soft tissues and oral cavity structures [5,42]. Thus, the periodontium was healthier in cases where the prosthetic treatment was accomplished using restorations manufactured by the CAD/CAM technic. This could have been due to the biomaterial rather than the manufacturing technique only [71,96]. Souza et al. [139] evaluated biofilm formation on various biomaterials used in prosthetic rehabilitation. No significant differences (*P* < 0.05) in absorbance and CFU/cm^2^ between biofilm growth on zirconia, porcelain, and titanium were found. A microbiological analysis related to microscopic examination discovered a higher accumulation of oral biofilms on Co-Cr-based biomaterials than on Ti or Zr, which are used for prosthetic constructions (Figure 5 and Figure 6).

Periodontal pathology around abutment teeth can be assessed with numerous new methods when fixed prosthetic restorations are performed. Oral metabolism and oral microflora, as well as the associations between them, have been investigated by Pei et al. [133]. They reported some possible biomolecules that could be used as valuable markers for preventive, prognostic, and individualized medicine in advanced chronic periodontal pathology. They demonstrated that the metabolic products and microorganisms in the GCF, together with clinical data exhibited a clear trend. The periodontal pathology could be reflected in the shift of the oral bacteria and the alteration in metabolic products in the GCF. A combination of both N-carbamylglutamate and citramalic acid produced reasonable accuracy for the prognostic diagnosis of widespread chronic periodontal pathology. Likewise, Oral Chroma™ was used to investigate the volatile sulfur compounds (VSCs) among subjects with temporary and permanent fixed dental restoration by Sinjari et al. [140]. They established that the Oral Chroma™ provides a complete evaluation of the VSCs as a diagnostic tool of oral malodor and that specialized oral hygienic procedures appeared to impact VSC production.

There has been considerable advancement in clinical dentistry [141,142,143,144,145]. With the use of advanced 3D digital technologies, teeth preparation can be evaluated, and the prosthesis can be fabricated accurately [146,147,148]. These help to minimize errors such as marginal gaps, discrepancies, etc. At present, restorative dentists are refining their practice and dental clinics are acclimatizing from conventional treatment methods to a digital workflow in the fabrication of dental prostheses [149]. Indeed, 3D printing techniques are now employed in developing simulators and models for dental education and the maintenance of prostheses. However, expertise is needed for the digital additive manufacturing of dental restorations used in prosthetic dentistry.

Finally, when planning a fixed dental prosthesis, adequate periodontal assessment and treatment, appropriate instructions, and motivation in self-performed plaque control as well as compliance to maintenance protocols, appear to be the most important factors to limit or avoid any potential negative effects on the periodontium caused by fixed prostheses [51,134].

## 9. Conclusions

Before starting prosthetic treatment, the condition of the periodontal tissues should be evaluated for their oral hygiene status, as well as the gingival and periodontal conditions. Determination of the gingival biotype, as well as the parameters of the GCF (amount, pH index, microbiological and cytomorphometric parameters), helps in the assessment and diagnosis of gingival and periodontal diseases as well as their successful management. Zirconium-based restorations made from the CAD/CAM technology provide better results, in terms of marginal fit, inflammation reduction, maintenance, and the restoration of periodontal health and oral hygiene, as compared to constructions made by conventional method and from other alloys. Compared to subgingival margins, the supragingival margins offer better oral hygiene, which can be maintained and does not lead to secondary caries or periodontal disease.

## Data Availability

Not applicable.
